# Chimeric Structure of Plant Malic Enzyme Family: Different Evolutionary Scenarios for NAD- and NADP-Dependent Isoforms

**DOI:** 10.3389/fpls.2018.00565

**Published:** 2018-05-11

**Authors:** Marcos A. Tronconi, Carlos S. Andreo, Maria F. Drincovich

**Affiliations:** Centro de Estudios Fotosintéticos y Bioquímicos, Universidad Nacional de Rosario, Rosario, Argentina

**Keywords:** phylogeny, malic enzyme, land plants, molecular evolution, NAD or NADP specificity

## Abstract

Malic enzyme (ME) comprises a family of proteins with multiple isoforms located in different compartments of eukaryotic cells. In plants, cytosolic and plastidic enzymes share several characteristics such as NADP specificity (NADP-ME), oxaloacetate decarboxylase (OAD) activity, and homo-oligomeric assembly. However, mitochondrial counterparts are NAD-dependent proteins (mNAD-ME) lacking OAD activity, which can be structured as homo- and hetero-oligomers of two different subunits. In this study, we examined the molecular basis of these differences using multiple sequence analysis, structural modeling, and phylogenetic approaches. Plant mNAD-MEs show the lowest identity values when compared with other eukaryotic MEs with major differences including short amino acid insertions distributed throughout the primary sequence. Some residues in these exclusive segments are co-evolutionarily connected, suggesting that they could be important for enzymatic functionality. Phylogenetic analysis indicates that eukaryotes from different kingdoms used different strategies for acquiring the current set of NAD(P)-ME isoforms. In this sense, while the full gene family of vertebrates derives from the same ancestral gene, plant NADP-ME and NAD-ME isoforms have a distinct evolutionary history. Plant *NADP-ME* genes may have arisen from the α-protobacterial-like mitochondrial ancestor, a characteristic shared with major eukaryotic taxa. On the other hand, plant *mNAD-ME* genes were probably gained through an independent process involving the Archaeplastida ancestor. Finally, several residue signatures unique to all plant mNAD-MEs could be identified, some of which might be functionally connected to their exclusive biochemical properties. In light of these results, molecular evolutionary scenarios for these widely distributed enzymes in plants are discussed.

## Introduction

Malic enzymes (MEs) catalyze the oxidative decarboxylation of L-malate, producing pyruvate, CO_2_, and NAD(P)H in the presence of a divalent cation (Mg^+2^ or Mn^+2^; Chang and Tong, 2003). MEs are part of a family of structurally related proteins (Malic Enzyme Family, MEF) that also includes malolactic enzymes (MLEs) and soluble oxaloacetate decarboxylases (OADs), which convert L-malate to L-lactate, and oxaloacetate (OAA) to pyruvate, respectively (Espariz et al., 2011; **Supplementary Figure S1**). MEs can be classified considering different criteria. Based on coenzyme specificity and their ability to decarboxylate OAA, MEs are divided into three categories: EC1.1.1.38 (NAD-dependent; decarboxylates added OAA), EC1.1.1.39 (NAD-dependent; does not decarboxylate added OAA), and EC 1.1.1.40 (NADP-dependent; decarboxylates added OAA; International Union of Biochemistry and Molecular Biology^1^; **Supplementary Figure S1**). Based on the NAD(P) binding domain, MEs can be further divided into two groups: Class I^2^ and Class II-MEs^3^. Class I groups MEs typically found in eukaryotes and in some eubacteria. This group also includes MLE of Gram-positive lactic acid bacteria (LAB). Otherwise, the Class II-MEs are almost exclusively constituted by archebacterial and eubacterial MEs, along with the OADs found in Gram-positive bacteria. In eukaryotes, Class II-MEs have been found to date only in the protist *Trichomonas vaginalis* and *Entamoeba histolytica* (Field et al., 2000; Dolezal et al., 2004). Class I and Class II-MEs group in two well-separated branches of a phylogenetic tree of MEF proteins (Espariz et al., 2011), indicating that that they do not share a common evolutionary history.

Malic enzyme is widely distributed in nature, being present in organisms of the major biological divisions. This is due to the extensive participation of its substrates and products in different metabolic pathways. The genome of higher animals (vertebrates) encodes a uniform set of three MEs, one cytosolic NADP-dependent enzyme (cNADP-ME, EC 1.1.1.40) and two mitochondrial isoforms: one NADP-dependent (mNADP-ME, EC 1.1.1.40) and another NAD-dependent (mNAD-ME, EC 1.1.1.38). Animal cNADP-ME and mNADP-ME provide NADPH for the biosynthesis of long-chain fatty acids or steroids (McKeehan and McKeehan, 1982; Goodridge et al., 1996), while mNAD-ME is only expressed in fast-growing and neoplastic tissues where it is implicated in energy metabolism (Moreadith and Lehninger, 1984). On the other hand, the genome of land plants (embryophytes) encodes a variable, species-dependent, ME set with several isoforms distributed among cytosol (cNADP-ME, EC 1.1.1.40), plastids (plNADP-ME, EC 1.1.1.40), and mitochondria (mNAD-ME, EC 1.1.1.39). Plant cNADP-MEs have been linked to plant defense responses (Schaaf et al., 1995; Alvarez et al., 2013), lignin biosynthesis (Schaaf et al., 1995), cytosolic pH control (Martinoia and Rentsch, 1994), regulation of stomatal closure (Outlaw et al., 1981; Laporte et al., 2002), and photosynthetic metabolism in Crassulacean acid metabolism (CAM) plants (Cushman, 1992). In plastids, plNADP-MEs are involved in the C_4_ photosynthetic process operating in the C_4_-NADP-ME subtype (Drincovich et al., 2010; Saigo et al., 2013) as well as in defense responses (Maurino et al., 2001; Chi et al., 2004) and lipid synthesis (Lai et al., 2002). In plant mitochondria, mNAD-ME participates in the photosynthetic process of C_4_-NAD-ME subtype species, but its constitutive presence in all tissues of C_3_ and C_4_ species indicates a housekeeping role for this isoform. In this regard, mNAD-ME could channel the large L-malate reserves commonly present in many plant species to the tricarboxylic acid (TCA) cycle for their complete oxidation (Tronconi et al., 2008) or participate in amino acid and lipid biosynthesis (Sweetlove et al., 2010).

Several biochemical and structural characteristics distinguish plant mNAD-MEs, e.g., the EC1.1.1.39 category is composed only of plant mNAD-MEs, as they are the only isoforms unable to decarboxylate OAA characterized to date. Interestingly, whereas the animal NAD(P)-MEs and plant NADP-MEs are assembled as homo-oligomers of monomers with molecular masses in the range between 60 and 66 kDa, plant mNAD-MEs can be structured as heteromers of two different subunits: α (˜60 kDa, or mNAD-ME1) and β (˜58 kDa, or mNAD-ME2) proteins, and also as α_2_ and β_2_ active homodimers (Willeford and Wedding, 1987; Tronconi et al., 2008, 2010b). In addition, these enzymes cannot catalyze the reverse reaction (reductive pyruvate carboxylation) as it has been described for animal NAD(P)-MEs and plant NADP-MEs (Mallick et al., 1991; Gerrard Wheeler et al., 2008; Tronconi et al., 2010a). Finally, the Arabidopsis α_2_ mNAD-ME homodimer displays a complex allosteric inhibition by L-malate not described for other MEs characterized up to this point (Tronconi et al., 2012, 2015). These properties of plant mNAD-ME, not shared with the other MEs found in higher eukaryotes, are puzzling and could have their origin in a different evolutionary history for these isoforms.

This work addressed the evolutionary relationship of Class I-MEs found in eukaryotes, focusing on the plausible divergent origin of plant mNAD-MEs. Multiple primary sequence alignments and modeling based on three-dimensional structures were explored using bioinformatic approaches to analyze elements exclusively present in plant mNAD-ME. The phylogenetic analysis provides support for different evolutionary scenarios for *NADP-ME* and *NAD-ME* genes in plants.

## Materials and Methods

### Retrieval of Sequences and Alignments

The sequences of MEs, MLEs, and OADs were recovered from the National Center for Biotechnology Information database^4^. For plants and algal species with entire genome information, MEs sequences were extracted from www.phytozome.net, while animal and fungal MEs were retrieved from www.genomesize.com and http://fungidb.org, respectively. BLASTP or TBLASN with a minimal *e*-value of 0.0001 was used to obtain homologous MEs in Chromista, Rhodophyta, Excavata, and Amoebozoa kingdoms with the human cNADP-ME (GI:EAW48664), human mNAD-ME (GI:EAW62981), and α subunit mNAD-ME from *Arabidopsis thaliana* (AT2G13560) used as seeds.

*Cyanophora paradoxa* and *Bigelowiella natans* ME sequences were retrieved from draft genomes (Curtis et al., 2012; Price et al., 2012) using TBLASN for searching. The partial nucleotide encoding sequences were translated and putative proteins were aligned by ClustalW for editing before evolutionary analyses.

For gene identification in *Rhizopus oryzae* and *Mucor circinelloides* with a potential photosynthetic ancestry, *Phytophthora sojae* genes, assumed to have a photosynthetic endosymbiotic origin (Tyler et al., 2006), were used as query to search in the genome database of *M. circinelloides*^5^.

For amino acid comparisons and general editing, sequences were aligned in a ClustalW format. Accession numbers of protein sequences used in the present study are provided in **Supplementary Table S1**.

### Prediction of Subcellular Localization of Proteins

Protein localization was analyzed using MitoProt II (Claros and Vincens, 1996), TargetP 1.1 (Emanuelsson et al., 2007), and ChloroP 1.1 (Emanuelsson et al., 1999). Sequences were considered as co-localized when at least two out of the three programs predicted matching results.

### Mutual Information Analysis

Co-evolving amino acid pairs in plant mNAD-ME isoforms were identified by the MISTIC (mutual information server to infer coevolution) method (Simonetti et al., 2013). Mature isoforms (without mitochondrial targeting peptide) from angiosperms were aligned and loaded at http://mistic.leloir.org.ar/index.php in a FASTA format. *A. thaliana* α subunit mNAD-ME (AT2G13560) was chosen as reference sequence. MISTIC calculated the Mutual Information (MI) between pairs of columns in the multiple alignments. Briefly, the frequency of appearance for each residue pair was compared with the individual observed frequency for each amino acid at the same positions (Buslje et al., 2009; Simonetti et al., 2013). Next, an MI score was calculated as the number of standard deviations that the observed MI value falls above the mean value obtained form a large set of permutated versions of the alignment for each pair position. MI scores higher than 6.5 provide sufficient specificity and sensitivity being the only ones reported (Buslje et al., 2009). Results were displayed in different formats (i) Circos representation for comparison through the full alignment of position pairs analyzed with MI, (ii) network graphs composed of nodes joined by edges, where nodes represent residues and edges between two nodes indicate an MI value > 6.5, and (iii) logo representation showing the amino acid enrichment and depletion for a group of MI connected nodes.

### Molecular Evolutionary Analyses

Multiple sequence alignments of amino acid sequences for evolutionary inferences were constructed with MUSCLE 3.2 version (Multiple Sequence Comparison by Log-Expectation; Edgar, 2004) implemented in MEGA6 software (Tamura et al., 2013) and manually edited for missing gaps or poorly aligned regions. The final ME alignment consisted of 142 sequences and 544 characters. Phylogenetic trees were inferred by the maximum likelihood (ML) and Bayesian inference (BI) methods via the MEGA6 (Tamura et al., 2013) and MrBayes 3.1.2 software (Ronquist and Huelsenbeck, 2003), respectively. In ML analysis, data samples were first analyzed to assess the best substitution model describing the observed sequence change. The goodness of fit of each model to the data was measured by the Bayesian information criterion (BIC) and the model with the lowest BIC score was considered the best description for a specific substitution pattern. For our data set, the lowest BIC value corresponded to the Whelan and Goldman model (WAG; Whelan and Goldman, 2001) using a discrete gamma distribution with five categories ([+G], α parameter = 2.0533) under the assumption that a certain fraction of sites are evolutionarily invariable ([+I], 2.6198% sites). Hence, this constituted the evolutionary model: WAG + G (2.0533) + I (2.6198). For ambiguous states and insertion–deletions, we chose a partial-deletion treatment with a site coverage parameter at 95%. The initial tree for the ML search was generated automatically by applying NJ and BIONJ algorithms, and its branch lengths were adjusted to maximize the likelihood of the data set for that tree topology under the selected model of evolution. Heuristic searches were conducted with the initial tree based on the nearest neighbor interchange (NNI) search where the alternative trees differ in one branching pattern. The final tree was the one whose arrangement of branches led to the highest ML value. Reliability of interior branches was assessed with 500 bootstrap re-samplings (MLB). Nodes with MLB values 50–69% were regarded as weakly supported, 70–84% as moderately supported, and 85–100% as strongly supported (Hillis and Bull, 1993). Phylogenetic trees were displayed using the MEGA software version 6.0 (Tamura et al., 2013). Substitutions per site are denoted as a separated bar with a number in each phylogenetic tree.

In the BI analysis, two parallel runs, each including four Metropolis-coupled Markov chain Monte Carlo (MC3) analysis, were run for 5,000,000 generations and sampled every 100 generations for each partition scheme. This generated an output of 50,000 trees. For an efficient Metropolis coupling, an incremental heating scheme of three heated chains and one cold chain in each run was used, with a temperature parameter setting of 0.08. In order to confirm that the chains achieved stationary, “burn-in” plots were evaluated by plotting log-likelihood against generation number. Also, the final average standard deviation of split frequencies was used as the convergence index, in which values < 0.01 indicated good convergence, and convergence of clade posterior probabilities within and between runs was checked using the potential scale reduction factor (PSRF). After determining convergence, the initial 25% of the sampled trees for each MC3 run were discarded as “burn-in,” and the post-burn-in trees were used to generate a 50% majority-rule consensus tree. The percentage of samples recovering any particular clade in a BI analysis represents the posterior probability (BPP) of a clade. Node with BPP values ≥95% were considered highly supported and ≤95% not supported. The tree figures were displayed using the FigTree v1.4 software^6^ and visually edited. Substitutions per site are denoted as a separated bar with a number in each phylogenetic tree.

### Identification of Signature Residues

Identification of well-conserved positions in the primary sequences that differ in other groups (SDP, specificity-determining positions) within a group of proteins was performed using SDPpred^7^. ME sequences belonging to A+V and G+R clades (see below) were aligned in a GDE (ClustalW) format before uploading. For each aligned column, the software computed a z-score for positions where the amino acid distribution is more closely associated with grouping than for an average position of the alignment, which is thus likely to be an SDP (Kalinina et al., 2004). Next, SDPpred evaluated the significance of the z-scores in order to decide if it is high enough to indicate an SDP. For this, SDPpred established a z-score threshold “*p*” (or B-cutoff) based on the computation of the Bernoulli estimator. Positions with z-score > *p* were designated as SDPs, as they were non-randomly generated (Kalinina et al., 2004).

## Results

### Amino Acid Sequence Analysis of Animal and Plant MEs

**FIGURE 1:**
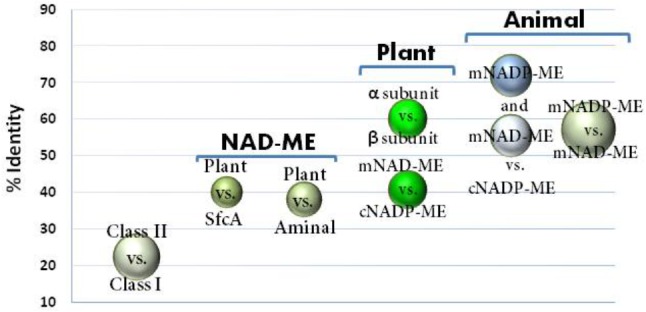
Sequence identity among ME isoforms comparing class I- versus class II-MEs; animal MEs; and relevant group isoforms from plant MEs and NAD-MEs. Circles indicate the identity values (%) obtained by ClustalW alignment among the different ME isoforms indicated in the graph. Circle size is proportional to the standard dispersion. For each comparison, 12 animal MEs and 20 plant ME sequences were used. For class I versus II comparison, ME sequences for 10 eubacteria, MaeB from *E. coli*, YtsJ from *Bacillus subtilis* (NC_000964.3), and the accessed protein WP_029552528 from *Solfolobus solfataricus* as representative sequences of Class II-ME were also included. The Class I-MEs used for calculations are denoted with (^∗^) in **Supplementary Table S1**. For clarity, comparison between plant cytosolic and plastidic NADP-ME was not included, which was centered on 85% identity.

**FIGURE 2:**
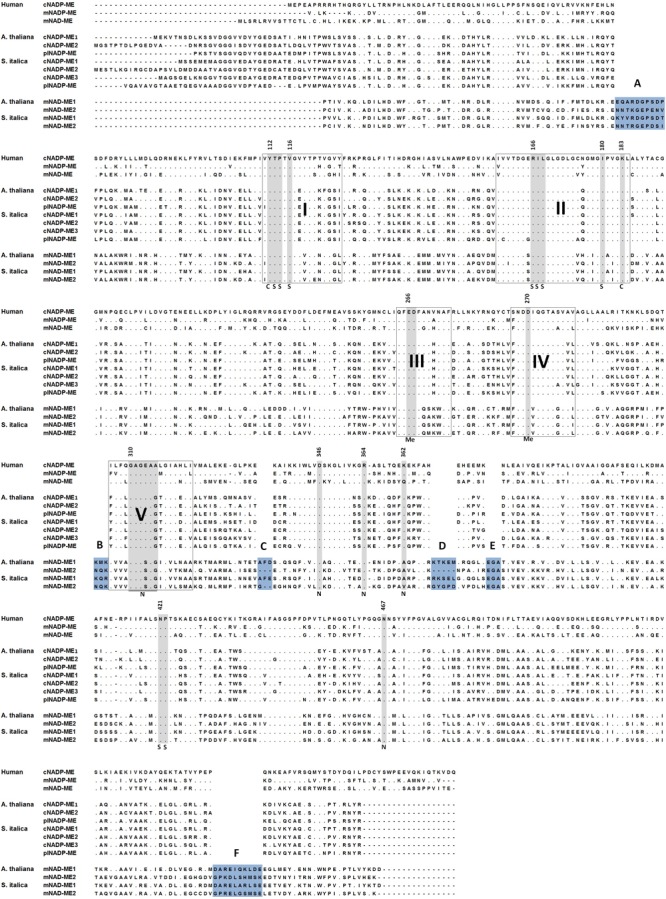
Amino acid sequence alignment of representative NAD-MEs and NADP-MEs. NADP-ME and NAD-ME sequences of human, *A. thaliana*, and *S. italica* as representative of animal, eudicot plants, and monocot plants, respectively, are shown. Conserved sequences found in all NAD(P)-ME (sites I–V) are highlighted. Insertions present in plant mNAD-MEs are highlighted in blue. Amino acid residues implicated in catalysis (C) as well as in metal (Me), L-malate (S), or cofactor (N) binding are highlighted in gray. Numbers for each amino acid residue refer to their position in human mNAD-ME primary structure.

Animal mNADP-ME and mNAD-ME share about 55% identity with each other and they are 72% and 55% identical to animal cNADP-ME, respectively (**Figure 1**). In plants, α and β mNAD-MEs are about 63% identical and share only 40% identity with cytosolic or plastidic counterparts (**Figure 1**). When animal and plant mNAD-MEs are compared, the proteins are nearly 38% identical. These relatively low identity values of plant mNAD-ME with isoforms from higher eukaryotes are similar to those found with the SfcA ME of *Escherichia coli* (**Figure 1**). Finally, Class I- and Class II-MEs, represented by the sequence from the archeon *Solfolobus solfataricus*, exhibit very low identity (about 20%) with each other (**Figure 1**). In this top-down analysis of sequences, plant mNAD-ME displays the five conserved sites previously defined for the ME family (Drincovich et al., 2001), including the two phosphate cofactor-binding signature motifs: site II and site V (**Figure 2**; Chang and Tong, 2003). A multiple protein sequence alignment of animal and plant NAD(P)-MEs reveals that the main differences in plant mNAD-ME lie in short amino acid insertions (indicated as A–F in **Figure 2**), with the amino (A) and carboxyl terminal (F) insertions being the most significant in extension. Two shorter insertions (B and C) are located near the second dinucleotide-binding signature motif that is part of the cofactor binding site. In addition, some single-amino acid substitutions in this region can be highlighted. For instance, the GAGEAA/G consensus sequence in animal NAD(P)-ME and plant NADP-ME changes to GAGSAG in plant mNAD-ME (**Figure 2**). Also, amino acid residues implicated in cofactor specificity are different in this enzyme. In NADP-ME, a Lys residue (K362 for human cNADP-ME) is the major determinant for NADP specificity (Hsieh et al., 2011). This amino acid is not present in animal or plant mNAD-ME; it has been substituted by Asn in animal mNAD-ME and by Ala or Val in plant mNAD-ME (**Figure 2**). Despite these differences, the residues that have been implicated in substrate binding and catalysis in human mNAD-ME are conserved in plant mitochondrial isoforms (**Figure 2**; Chang and Tong, 2003).

### Co-evolutionary Residue Positions

In view of the significant changes at the primary sequence level of plant mNAD-ME, we evaluated the extension of the co-evolutionary relationships between amino acid positions through the MI principle. MI assumes that mutations of essential residues may occur only if a compensatory mutation elsewhere in the protein also takes place to preserve activity (Martin et al., 2005). Pairs of residues in a sequence alignment exhibiting high MI score values are postulated to have coevolved and thus are probably functionally connected. Since over 100 aligned sequences are required for an accurate MI prediction and such a number of plant mNAD-MEs are not currently available, our results are only an approximation to the inference of the co-evolutionary scenario. Data sets of 78 α and β plant mNAD-ME mature sequences (590 residues long) were fully aligned and MI calculation was performed by MISTIC (Simonetti et al., 2013). High-scoring MI pairs were accumulated in three main regions of α and β plant mNAD-ME: 53–71, 317–376, and 547–583 (**Supplementary Figure S2A**). Individual residues with a high number of MI connections can be found within these regions. Interestingly, many of these residues are part of the A, C, D, and F insertion sequences, which are exclusive to plant mNAD-ME (**Supplementary Figure S2A**). For example, residues in the A and F regions, which are far apart in the primary sequence, were highly MI interconnected with each other and with some residues of insertions C and D (**Supplementary Figure S2B**). This suggests that these unique sequence segments could be relevant for plant mNAD-ME functionality. Additionally, we analyzed pairs of α and β sequences fused in an amino to carboxyl arrangement in both α–β and β–α combinations. A common number of MI connections involving residues belonging to the two subunits were identified for both combinations (**Supplementary Figure S2C**).

### Gene Structure of Animal and Plant MEs

The structure of vertebrate and plant *ME* genes was analyzed considering the number and homology of encoding exons. *ME* genes of four animal species that belong to different taxonomic classes were analyzed: *Homo sapiens* (Mammalia), *Xenopus laevis* (Amphibia), *Danio rerio* (Actinopterygii), and *Columbus livia* (Aves). The structure of *NAD(P)-ME* genes is well conserved in the four animal species, with c*NADP-ME* and *mNADP-ME* genes containing 14 encoding exons, and *mNAD-ME* 15 exons (**Supplementary Figure S3A**). In each species, all exons of the three *ME* genes are homologous and the extra exon present in *mNAD-ME* arises from the split of exon number 12 of c*NADP-ME* or *mNADP-ME.* Thus, all the animal *ME* genes seem to be evolutionarily related.

Among plants, we analyzed the complete *ME* gene set in the bryophyte *Physcomitrella patens* and three tracheophytes species: *Selaginella moellendorffii* (Lycopodiophyta), *A. thaliana* (Angiospermae, eudicot), and *Zea mays* (Angiospermae, monocot), which are distant species and show different sets of *ME.* In general, plant *ME*s exhibit higher numbers of encoding exons than the animal genes with some degree of species-dependence (**Supplementary Figure S3B**). In this regard, both *NADP-ME* and *NAD-ME* genes present 19 encoding exons, except *cNADP-ME2* of *Z. mays* with eight, since exons 12–19 are fused. Although there is some variability in the number and structure of exons between animal and plant *NAD(P)-ME* genes, two conclusions may be drawn from gene conformation analysis. First, some degree of colinearity between the exons of plant *NADP-ME* genes and those from animals can be noted. Second, each encoding exons from a particular *cNADP-ME* or *plNADP-ME* gene in plants has its counterpart in other *cNADP-ME* or *plNADP-ME*, but not in *mNAD-ME* genes, where their exons only show homology to each other (**Supplementary Figure S3B**).

### Phylogeny Pattern of Plant NADP and NAD-ME

**FIGURE 3:**
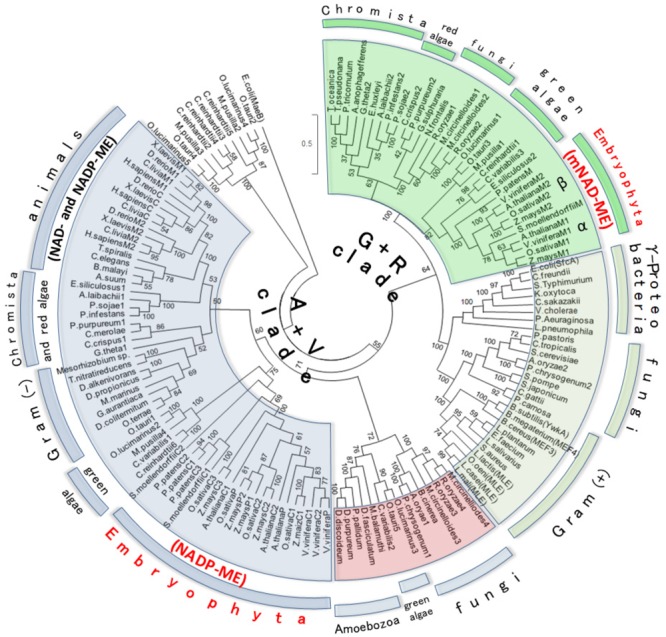
Maximum likelihood tree of Class I-ME protein sequences. Evolutionary history was inferred by using the ML method based on the WAG + G (2.0533) + I (2.6198) evolutionary model. The tree with the highest log likelihood (–73118.4895) is shown. Analysis involved 134 amino acid sequences (see **Supplementary Table S1** for list of gene IDs). All positions with less than 95% site coverage were eliminated. The final data set comprised a total of 491 positions. The tree is drawn to scale, with branch distance separating two taxa measured in the number of substitutions per site. Numerals indicate support of branches, which were assessed using 500 boostrap replicates. MaeB sequence from *E. coli* (a Class II-ME) was included in the analysis as out-group. For animal and plant MEs, M, P, and C indicate mitochondrial, plastidic, and cytosolic isoforms, respectively. The A+V Clade groups Animalia + Viridiplantae MEs, while G+R Clade clusters green + red isoforms. Plant ME family members, which are distributed between A+V and G+R lineages, are highlighted in red letters. α and β indicate the two plant mNAD-ME isoforms.

The above results suggest that plant mNAD-MEs could be evolutionarily unrelated to other eukaryotic MEs. Thus, to address the evolutionary relationship of plant mNAD-ME, we performed phylogenetic analyses using Class I-MEs amino acid sequences from organisms that belong to the major taxonomic divisions: Archaeplastida (Glaucophyta, Rhodophyta [red algae], and Viridiplantae [green algae and Embryophyta]), Opisthokont (Animalia and Fungi), Chromista (Crytophyta, Heterokonta, and Haptophyta), Rhizaria (Chlorarachniophyta), Amoebozoa, and Eubacteria (Gram-negative and Gram-positive bacteria). Archeal ME and eubacterial OAD sequences were excluded from our analyses as they are members of the highly divergent Class II-ME group. Hence, data sets of 134 eubacterial and eukaryotic MEs were retrieved from databases, aligned and, after exclusion of ambiguously aligned regions, 491 amino acid positions were included in ML and BI phylogenetic analyses (see **Supplementary Table S1** for list of gene ID’s). When two sequences showed >85% of similarity, one of them was excluded from the alignment. Eukaryotic Class I-MEs grouped in two major clades in the ML tree (**Figure 3**). One clade contains all animals NAD(P)-MEs along with cNADP-MEs and plNADP-MEs from plants. This group also contains some algae and chromist sequences and thus constitutes the “Animalia + Viridiplantae clade” (A+V clade). Homologous sequences from the paraphyletic Gram-negative group, comprising Verrucomicrobia (*Opitutus terrae*) and α-Proteobacteria (*Mesorizhobium* sp.), are also included in the A+V clade. Amoeba sequences branch as a subgroup along with fungal and algae enzymes in this clade. On the other hand, plant mNAD-ME conform the second major eukaryotic clade together with sequences of green and red algae and chromists (**Figure 3**). We named this grouping the “Green + Red clade” (G+R clade) because in addition to embryophytes, it comprises green and red algae, as well as organisms that are evolutionarily related with them through secondary endosymbiosis. The G+R clade contains a sister group of MEs from Gram-negative and Gram-positive bacteria, and some fungal sequences. Interestingly, all Gram-negative sequences in this group belong to γ-proteobacteria class. MLEs are also included in this subclade, as they nest within Gram-positive MEs (**Figure 3**). Finally, some green algae ME isoforms constitute a cluster separated from the remaining sequences by a very long branch. The analysis of these sequences indicates several mutations at positions of the conserved motifs that characterize the ME family (not shown), suggesting that they could be a case of neofunctionalization in the MEF. The BI tree topology was highly congruent with those deduced by ML analysis (**Supplementary Figure S4**) with branches well resolved and supported (BPP ≥ 95%). In particular, branches grouping the deep lineages of ME, which were weakly/moderately sustained in ML approach, were strongly supported in the BI analysis. When comparing ML and BI trees, two mayor differences can be found: amoeba, fungi, and algae sequences form a third group of eukaryotic ME, separated from the A+V clade in the BI tree and Gram-negative sequences do not nest with animal isoforms, constituting a simple cluster within the A+V clade (**Supplementary Figure S4**). It should be mentioned that the highly divergent algae ME sequences were omitted in the BI analysis.

**FIGURE 4:**
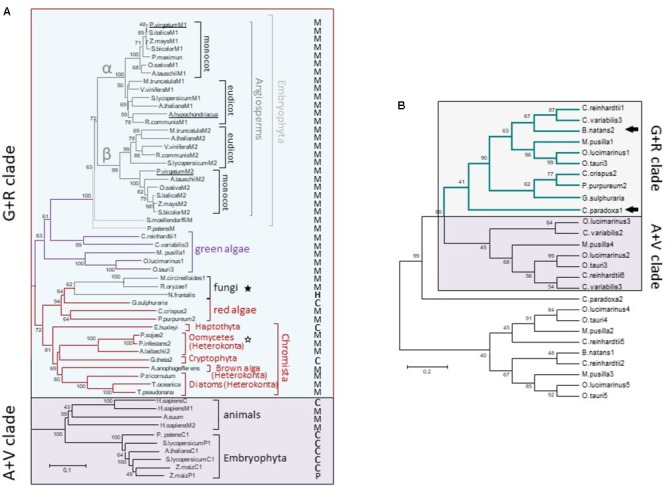
Phylogenetic tree of G+R clade of Class I-ME **(A)** and inclusion of *C. paradoxa* and *B. natans* MEs **(B)**. **(A)** Maximum likelihood tree of ME sequences focusing on the G+R clade. Methods are the same as in **Figure 3** [WAG + G(2.0533) + I(2.6198) model]. The tree is drawn to scale, with branch lengths measured in the number of substitutions per site. M, P, H, and C indicate mitochondrial, plastidic, hydrogenosomal, or cytosolic location, respectively. Stars indicate non-photosynthetic organisms with proved (empty stars) or not proved (filled stars) photosynthetic past. **(B)** Maximum likelihood tree constructed (WAG model) with 240 amino acid ME sequences from green and red algae along with the available translated protein regions from the glaucophyte *Cyanophora paradoxa* and the secondary photosynthetic *Bigelowiella natans* (see **Supplementary Table S1** for details). Arrows indicate *C. paradoxa* and *B. natans* sequences included into the G+R clade. In both trees, numerals indicate support of branches with 500 boostrap replicates.

Although the monophyly of the G+R clade is consistently recovered and strongly supported by ML analysis, internal branches corresponding to some chromist sequences were moderately supported (**Figure 3**). This could be due to the existence of long branches in the extensive phylogenetic analyses that destabilize the otherwise robust grouping (Swofford et al., 1996). Longer branches were indentified for green algae MEs belonging to the A+V clade and for Gram-positive MLEs (data not shown). Thus, a new ML tree mainly focused on G+R sequences, including a larger number of mNAD-ME sequences from C_3_ and C_4_-NAD-ME subtype plants, was constructed. In the absence of long branches, the MLB support for chromists increased significantly (**Figure 4A**).

For α and β mNAD-ME subunits of Angiosperms, monocot and eudicot isoforms are grouped separately in the G+R clade (**Figure 4A**). Unlike Angiosperms, the moss *P. patens* and the ancient tracheophyte *S. moellendorffii* possess a single mNAD-ME isoform, which constitutes the most immediate out-group from the monocot/eudicot grouping and completes the Embryophyta cluster in the phylogenetic tree. Some green algae sequences nest closely to embryophytes. Here, the isoforms of both freshwater green algae (*Chlamydomonas reinhardtii* and *Chlorella variabilis*) and planktonic green algae (*Ostreococcus* spp. and *Micromonas pusilla*) were predicted to be located in the mitochondria (**Figure 4A**). Red algae and chromist sequences constitute a closed and highly supported subgroup in the G+R clade, where mitochondrial allocation was predicted for almost all MEs except for the red alga *Galdieria sulphuraria*, the haptophyte *Emiliania huxley*i, and the crytophyte *Guillardia theta* (**Figure 4A**). Interestingly, a reduced fungal clade composed by *Neocallimastix frontalis, R. oryzae*, and *M. circinelloides* was found to nest within red algae sequences with very strong bootstrap support. MEs from *R. oryzae* and *M. circinelloides* are possibly located in the mitochondria while the *N. frontalis* isoform would be targeted to the hydrogenosome, a mitochondria-related compartment present in this anaerobic fungus (van der Giezen et al., 1997). Along with Oomicetes, these fungi constitute a group of non-photosynthetic organisms in the G+R clade (**Figure 4A**). But, unlike Oomicetes, they lack a proved endosymbiotic evolutionary relationship with photosynthetic organisms. Even so, the position of *R. oryzae* and *M. circinelloides* MEs in the red algae sub-cluster is supported by the finding that other genes in these species are more related to homologs found in red algae and cyanobacteria than in fungi (**Supplementary Figure S5**).

Finally, we tracked a G+R-type ME in an ancient Archaeplastida member, the glaucophyte *Cyanophora paradoxa*, as well as in the chlorarachniophyte *B. natans*, a secondary (engulfed green alga) photosynthetic alga. For these species, the ME sequences are not complete and thus, a new tree using 240 aligned amino acid positions along with those corresponding to red and green algae homologs was constructed. Both *C. paradoxa* and *B. natans* possess at least one ME isoform phylogenetically related to the algal proteins that are members of the G+R clade (**Figure 4B**).

### Signature Residues of the G+R Clade

**FIGURE 5:**
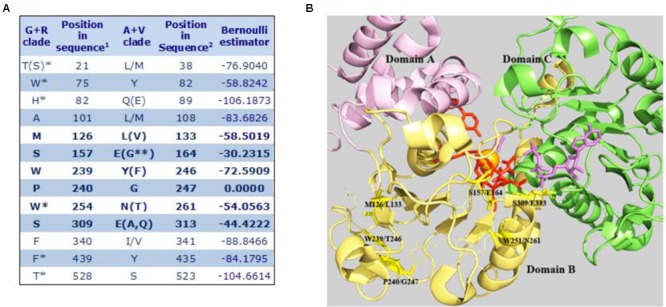
Specificity-determining position in MEs of the A+V and R+G clades. **(A)** ^1^Amino acid positions in the primary sequence of α mNAD-ME from *A. thaliana*. ^2^Amino acid positions in human mNAD-ME. Residues between parentheses show a frequency of occurrence lower than 10%. Residues on the right side of bars show a frequency of occurrence higher than 10%. ^∗^Corresponds to SDPs identified only when embryophyte G+R sequences were compared with all A+V groupings. ^∗∗^Corresponds to one residue found only in plastidic NADP-ME from *Setaria italica.* Letters in bold indicate SDPs with the highest z-scores that were defined as signature residues. **(B)** The SDPs with the highest scores are mapped onto the three-dimensional structure of the human mNAD-ME complex with the L-malate analog (oxalate, in magenta), NAD^+^ (in magenta), Mn^+2^ (in orange), and fumarate (not shown; 1GZ3, Yang et al., 2002). Signature residues are shown as stick yellow lines. Residues involved in L-malate binding and catalysis are shown as stick red lines. Residues contacting signature residues are shown as fine lines. Domains A, B, and C are shown in different colors. For clarity, whole Domain D and the αB3-helix of Domain B were omitted in the model.

Based on the aforementioned evolutionary relationship, MEs found in higher eukaryotes are included in two groups, the A+V clade and the G+R clade. We used SDPpred (specificity determining position prediction; Kalinina et al., 2004) to search for residue positions that are well conserved within these two groups, but differ between them, in order to assign a signature amino acid sequence to each clade. Two different SDP predictions were performed: one considering all eukaryote A+V and G+R sequences and another one restricted only to the embryophyte G+R grouping (plant mNAD-MEs) with all eukaryote A+V MEs. The 13 SDPs exhibiting the highest z-scores considering both analyses are shown in **Figure 5A**. Twelve SDPs showed an invariable amino acid residue for MEs that belong to the G+R clade but only five unambiguous positions were predicted for A+V sequences. Using the tridimensional structure of human mNAD-ME (Yang et al., 2000), we observed that the six SDPs with the highest z-cores are located near the active site, as the farthest SDP was located at 8Å from the metallic center (**Figure 5B**). In this regard, using the α mNAD-ME from *A. thaliana* as a reference sequence, we found that residues S157 and W254 conserved in G+R sequences change to E164 and N261, respectively, in the A+V grouping. These residues are located at the active site in human mNAD-ME (**Figure 5B**). Particularly, S157 is located in the first cofactor-binding signature motif (Site II in **Figure 2**). Also, residues M126, W239, and P240 of the G+R group are almost invariably replaced by L133, Y246, and G247 in the A+V sequences, respectively, and they are part of domain B where the active site is located (**Figure 5B**). In particular, when residue P240 of the G+R group is replaced by G, this site is predicted to be an SDP with the highest confidence (*p*-value = 0.000; **Figure 5A**). Finally, S309 in the G+R group is replaced by G313 in the A+V grouping, a highly conserved residue located in the second dinucleotide-binding signature motif (Site V in **Figure 2**).

## Discussion

### Molecular Evolution of Plant mNAD-MEs

Plant mNAD-MEs show the lowest identity (38–40%, **Figure 1**) and similarity (57–60%, not shown) values when compared with either plant NADP-MEs or NAD(P)-MEs from distantly related organisms (animal or *Escherichia coli*). These numbers represent the minimum homology values shared among sequences that belong to Class I-MEs. In this regard, when Class I- and Class II-MEs are compared, the identities are roughly 20% (**Figure 1**), and conserved residues are only those implicated in catalysis (not shown). Despite having substantial sequence diversity, MEs share a similar overall tertiary architecture (Chang and Tong, 2003). In fact, the NAD(P)-binding Rossmann fold domain that is part of the MEF structure has been denoted as an extreme case where folding is non-sequence driven (Bhattacharyya et al., 2012). Thus, an important flexibility in sequence seems to exist in the MEF and, as an example of this property, plant mNAD-MEs posses several short amino acid residue insertions distributed throughout the primary structure (**Figure 2**). The presence of such segments can be traced to the lower plants *P. patens* and *S. moellendorffii* as well as to green algae, but not to red algae or chromists (not shown), suggesting that they were gained after the split of the green and red algal lineages. Co-evolution analysis of residue pairs indicated that these segments exclusive to plant mNAD-ME could be important for enzymatic functionality, since they are located in MI networks (**Supplementary Figure S2**). Crucially, as a common number of MI, connections involve residues belonging to different subunits (**Supplementary Figure S2C**), these inter-subunit co-evolutionary pairs could be the basis of the exclusive heteromeric assembly that characterizes plant mNAD-MEs. Although these regions are far away from each other in the primary structure, co-evolving insertions might be close in folding intermediates or might be part of a transmission signal pathway connecting allosteric and active sites or subunit interfaces (Goodey and Benkovic, 2008; Marino Buslje et al., 2010). In any case, it is clear that a requirement to maintain a particular function constraints structural diversity in these exclusive regions of plant mNAD-ME.

### *NADP-ME* and *NAD-ME* Genes Were Independently Acquired in Plants

**FIGURE 6:**
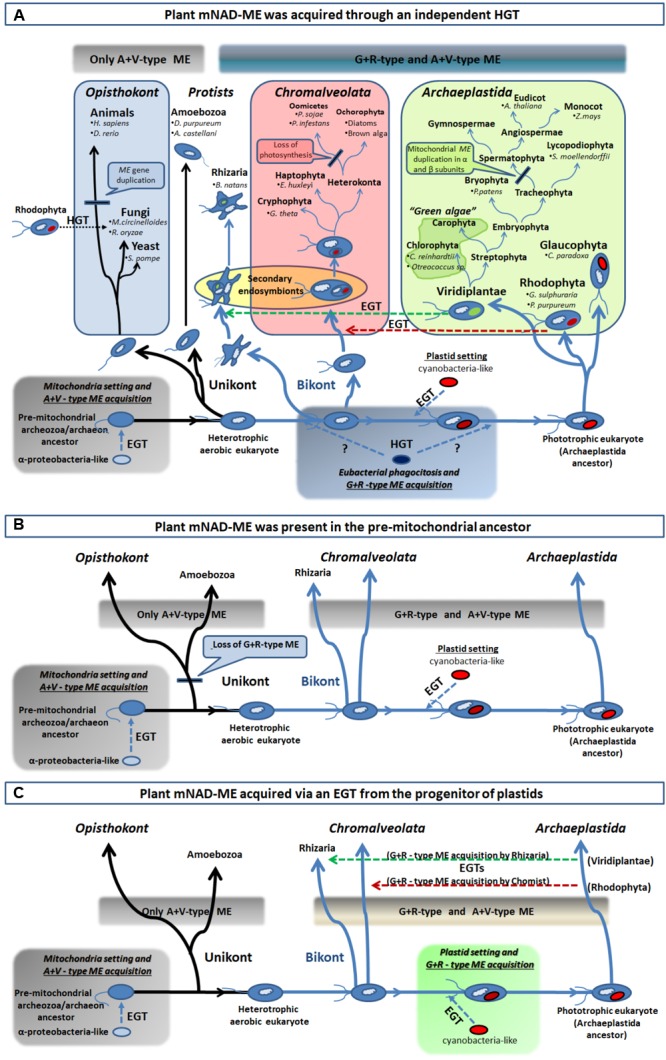
Different scenarios of acquisition of the R+G clade of Class I-ME in eukaryotes. **(A)** From bottom left, an extensive EGT from an engulfed α-proteobacteria-like ancestor (mitochondria precursor) to the nucleus of a unikont (one flagellum) host took place. This resulted in the establishment of eubacterial ME version in early eukaryotes and the foundation of the A+V clade of ME. After unikont and bikont (two flagella) splitting and prior – or post – plastid setting (indicated by question marks), an HGT event from one eubacteria (possibly a γ-proteobacteria-like) into the Archaeplastida ancestor established the G+R clade of Class I-MEs in eukaryotes. This allowed spreading of G+R isoforms in all primary photosynthetic organisms, as well as, in the secondary phototrophs (Chromalveolata and Rhizaria) via an EGT (green and red horizontal broken line arrows) and in some fungi by an HGT event (black horizontal dotted line arrow). **(B)** The G+R ME could be the ancestral isoform that was present in the pre-mitochondrial, and founder of the eukaryotic lineage, ancestor (the “Archezoa,” or an autotrophic methanogenic archaeon, depending on the model for the evolution of eukaryotes). The establishment of mitochondria in this ancestor allowed the acquisition of an additional isoform (the type A+V) and this chimeric structure for the MEF was maintained by the bikont lineage. However, only the A+V-type ME was retained by the unikont ancestor, as it lost the more ancestral isoform in what could have been a genetic replacement. **(C)** The G+R ME acquisition by photosynthetic organisms (containing the A+V ME derived from the mitochondria setting) may have taken place in with the plastid genesis, through the extensive EGT from the engulfed cyanobacteria to the host genome. Hence, the presence of this isoform in secondary phototrophs organisms results from secondary endosymbiosis processes, as is explained in **A**. For clarity, the major taxa of eukaryotic organisms were omitted in **B** and **C**.

A better understanding of the particular molecular evolution of plant mNAD-MEs, on which their exclusive biochemical properties rely can be achieved through the comprehensive phylogenetic approach performed in this work. ML and BI analyses indicate that Class I-MEs from eukaryotic organisms are separated in two principal branches (**Figure 3** and **Supplementary Figure S4**). The A+V cluster and its immediate out-group include sequences of all major taxa, from Amoebozoa to Embryophyta along with ME from Gram-negative bacteria (**Figure 3**). Because of their presence in unrelated organisms and the inclusion of α-proteobacteria sequences into the clade, we conclude that eukaryotes acquired A+V isoforms from the α-proteobacterial-like ancestor of mitochondria through the extensive endosymbiotic gene transfer (EGT) that took place when this organelle was established (Keeling and Palmer, 2008; **Figure 6**).

On the other hand, the G+R clade is almost solely constituted by primary and secondary photosynthetic organisms and species displaying photosynthetic metabolism in the past (**Figures 3, 4A**). Since the presence of G+R isoforms can be traced to the primitive photosynthetic endosymbiont, the glaucophyte *C. paradoxa* (**Figure 4B**), and considering that most of them are predicted to have a targeting peptide (**Figure 4A**), the incorporation of this isoform in mitochondria must have been an early event during evolution of the Archaeplastida lineage. Unlike the A+V clade, eubacterial MEs do not nest within the G+R group, but γ-proteobacteria sequences branch close to it with moderate (MLB = 64%) and strong (BPP = 98%) support (**Figure 3** and **Supplementary Figure S4**). The α-proteobacterial MEs were not closely related to the γ-proteobacteria clade, which indicates that it is unlikely that G+R type MEs were gained via mitochondrial endosymbiosis. Also, except for a very small group of related fungi (**Figures 3, 4A**), the lack of G+R MEs in other non-photosynthetic eukaryotes lineages further reduces the likelihood of a mitochondrial origin. Given these data at hand, one possible scenario would be that G+R-type MEs were established in mitochondria of photosynthetic organisms after unikonts and bikonts split via an independent horizontal gene transfer (HGT), possibly from a γ-proteobacteria-like to the Archaeplastida ancestor genome (**Figure 6A**). At this point, it is possible that the presence of a mitochondrial G+R-type ME was a precondition for plastid establishment or, alternatively, it was a subsequent requirement for the success of the emerging photosynthetic eukaryote (**Figure 6A**). Thus, if the G+R-type ME was implanted in the Archaeplastida ancestor through an HGT event, the presence of this kind of ME isoform in mitochondria of secondary photosynthetic organisms, chromalveolates and rhizarians, should be the result of the implantation of a red or green alga, respectively, in two different heterotrophic ancestors (Qiu et al., 2013; **Figure 6A**).

The acquisition of enzymes by the Archaeplastida ancestor through HGT has been proposed as a common phenomenon in this single-cell phagotrophic organism that allowed the rapid spreading of the lineage (Keeling and Palmer, 2008; Ball et al., 2013; Qiu et al., 2013), resulting in a more rapid change in biochemical diversity and metabolic versatility than evolution of isoenzymes by gene duplication and subsequent neofunctionalization (Ghoshroy et al., 2010). A γ-proteobacterial origin was proposed for a subgroup of Class II plant glutamine synthetase (GSII_B_) and phosphoenolpyruvate carboxylase (PEPC) genes (Sanchez and Cejudo, 2003; Ghoshroy et al., 2010). For GSII_B_, the timing of the HGT event was estimated when establishing the Archaeplastida linage (Ghoshroy et al., 2010). Hence, it is possible that the three genes could have the same evolutionary history in plants.

It is worth to mention that this is not the only possibility for the origin of mitochondrial G+R-type ME, as our phylogenetic results can also be explained by at least other two, albeit less parsimonious, scenarios (**Figures 6B,C**). Without considering the model for the evolution of eukaryotes we adopt, “the archezoa hypothesis” or “the symbiogenesis hypothesis” (Gray, 2012; Archibald, 2015), one other possibility would be that the G+R-type ME could constitute the original isoform present in the pre-mitochondrial ancestor (**Figure 6B**). Because almost all the extant non-photosynthetic organisms lack the G+R ME (**Figure 3**), this isoform must have been lost very early in eukaryotic evolution, probably involving the Unikot lineage (last eukaryote common ancestor of Opisthokont and Amoebozoa) that only kept the A+V ME derived from the EGT. Contrary, G+R ME was conserved in the Bikont lineage (an early ancestor of Archaeplastida), and then, their photosynthetic descendants present both G+R and A+V MEs (**Figure 6B**). However, the observation that none of the current Archaea species (the prokaryotes most closely related to eukaryotes) exhibit Class I-ME reduces the likelihood of this scenario. The second possibility for the presence of mitochondrial G+R-type ME in all photosynthetic organisms connects with the chloroplast evolution; this isoform could have been gained through EGT between the cyanobacterial progenitor of plastids and the heterotrophic host (**Figure 6C**). However, the genome analysis of extant cyanobacteria has not uncovered Class I-MEs sequences (Espariz et al., 2011), which argues against this possibility. Despite their inconsistencies, both scenarios still maintain a certain degree of plausibility, considering that, although neither Archaea nor cyanobacteria genomes encode a G+R-type ME to date, they could have done so in the past.

Besides, our results also suggest that eukaryotes from other kingdoms used independent strategies for acquiring the current set of NAD(P)-ME isoforms. While invertebrates hold a single nuclear *NAD-ME* gene, the gene family coding the full set of the cytosolic and mitochondrial NAD(P)-MEs is derived from a α-proteobacteria-related ancestral gene in vertebrates (**Figure 3**). In this case, modifying cofactor affinity (NADP/NAD) must have been a highly plausible evolutionary event, given the limited amino acid determinants required to completely switch the dinucleotide specificity (Hsieh et al., 2011). Even in land plants, the plastidic NADP-ME isoforms were generated by duplications of genes encoding cytosolic isoforms, which in turn are related to α-proteobacterial progenitors of mitochondria (**Figure 3**). On the other hand, it seems that fungi adopted uncommon mechanisms for gaining mitochondrial ME, as sequences clustered in several branches of the phylogenetic tree (**Figure 3**). Some fungi possibly duplicated a pre-existing gene or changed the subcellular location of the genetic product, while others attained it through HGT events with Eubacteria (**Figure 3**) or even with red algae, such as *M. circinelloides* and *R. oryzae* (**Supplementary Figure S4**). This evolutionary repertoire of fungal MEs is consistent with recently reported studies (Vorapreeda et al., 2013). Another example of an independent mitochondrial ME acquisition in eukaryotes is the case of the amoeba *E. histolytica*. In this case, it is postulated that the hydrogenosomal Class II-ME isoform was obtained from an archaeon related to *Archaeoglobus fulgidus* (Field et al., 2000).

Therefore, plant mNAD-ME seems to be unrelated to cNADP-ME and plNADP-ME counterparts, having been acquired in an independent evolutionary process. The different intron–exon constitution of *NADP-ME* and *NAD-ME* genes is in good agreement with this notion (**Supplementary Figure S2**). If both gene classes had been incorporated into the plant genome in different stages of evolution, then they would have been subjected to independent intron gain events. A chimeric structure has also been reported for several gene families encoding enzymes of the TCA cycle, as citrate synthase, aconitase, isocitrate dehydrogenase, and malate dehydrogenase (Cavalcanti et al., 2014). In fact, phylogenetic studies for the enzymes of the glycolysis, TCA cycle, gluconeogenesis, and “Calvin–Benson” cycle, among other, indicated that the pathways evolve as coherent entities of enzymatic activity rather than as protein associations (Schnarrenberger and Martin, 2002). Moreover, the pathway-associated genes can be easily replaced by equivalents through lateral gene transfer to furnish to the pathway new functions to full fit specific physiological demands. This implicates that a gene family, with several isoforms differing in their subcelluar location and/or being involved in unrelated processes in different organisms, can exhibit an evolutionary-based organism-specific composition.

### SDP Residues Could Be Associated to the OAD Reaction

We found several SPD residues that could determine the inclusion of an ME sequence into one particular phylogenetic group (**Figure 5B**). The fact that the more constrained analysis, limited to embryophytic G+R isoforms, resulted in the incorporation of only one high z-score SDP (W254) suggests that signature residues are a footprint of the whole phylogenetic group. In this regard, residues homologous to W239 and S309 are present in current γ-, but not α-proteobacteria, bolstering our idea that G+R-type ME origin was not associated with mitochondrial biogenesis. Regarding S309, this residue as well as S157 is part of the consensus sequence GXGXXG/A included in the two phosphate cofactor-binding regions (**Figure 2**). Members of the G+R clade show an invariant consensus sequence GXGSXG/A in both regions, but in the A+V isoforms, the Ser residue is not present, being mainly substituted by Glu(**Figures 2, 5B**).

Interestingly, we observed that six residues with the highest SDP scores form a cluster of spatial positions surrounding the active site (**Figure 5B**). Because the evolution-based division separates those animal and plant MEs able to decarboxylate OAA (included in the A+V clade) from those that cannot (plant mNAD-ME in the G+R clade), it is exciting to suppose that some of these catalytic site-linked SDP residues could have a role in the ability of the ME to catalyze the secondary OAD reaction. Also, taking into account the footprint nature of relevant SDP residues in the G+R clade (**Figure 5A**), the lack of OAA decarboxylation activity would be an emergent property of all G+R isoforms positively selected during evolution of the photosynthetic eukaryotic linage. A site-directed mutagenesis approach where SDP residues are replaced will shed light on this sequence-function relationship in plant mNAD-MEs.

## Conclusion

The MEF exhibits substantial sequence diversity, albeit the related overall tertiary architecture is conserved in the resolved crystallographic structures of several ME members. By adopting a not highly dependence on sequence fold, MEF members have undergone a sub- and/or neo-functionalization during evolution, exemplified by the OAD and MLE activities, as well as the lack of OAD and reverse reaction activities of plant mNAD-MEs. Also, this ability also accounts for the broad range of kinetic parameters, cofactor specificity [NADP(H) and/or NAD(H)], multimodular conformation (e.g., MaeB from *E. coli*), and the remarkable divergence in the nature of allosteric compounds regulating enzymatic activity (Teller et al., 1992; Bologna et al., 2007; Gerrard Wheeler et al., 2008; Tronconi et al., 2010b; Alvarez et al., 2013). Despite this, certain restrictions were imposed on A + V MEs sequence evolution that prevented their co-optation to address the mitochondrial metabolism of photosynthetic organisms. Hence, as in a “*shuffle and deal again*” scheme, the coupled-photosynthesis evolution molded a particular mNAD-ME structure with exclusive biochemical characteristics. The possibility that some SDP residues of G+R MEs are functionallyconnected to the biochemical properties of these isoforms would be an example of such “tailor-made” mechanisms that relied on the flexibility of certain sequence folds in the MEF.

## Author Contributions

MT designed the concepts and performed the bioinformatic approaches of this study. MT and MD were involved in the analysis and interpretation of the results. MT, in collaboration with the other authors, drafted the manuscript.

## Conflict of Interest Statement

The authors declare that the research was conducted in the absence of any commercial or financial relationships that could be construed as a potential conflict of interest. The reviewer SR and handling Editor declared their shared affiliation.

## Footnotes

1 http://www.chem.qmul.ac.uk/iubmb

2 https://www.ncbi.nlm.nih.gov/Structure/cdd/cddsrv.cgi?uid=133454

3 https://www.ncbi.nlm.nih.gov/Structure/cdd/cddsrv.cgi?uid=133453

4 http://ncbi.nlm.nih.gov

5 http://genome.jgi-psf.org/Mucci2/Mucci2

6 http://tree.bio.ed.ac.uk/software/figtree

7 http://bioinf.fbb.msu.ru/SDPpred

## Supplementary Material

FIGURE S1 Reactions catalyzed by MEF proteins. ME and MLE are involved in the conversion of L-malate into pyruvate and L-lactate, respectively. OAD enzymes catalyze the decarboxylation of OAA to give pyruvate. In the MLE catalysis, NAD acts as cofactor removing two electrons from L-malate and transferring them to pyruvate. Dotted arrow in MLE reaction indicates that the reverse activity has not been measured to date. The presence of a divalent cation (Me^2+^) is required in all cases.

FIGURE S2 Co-evolutionary residue positions of plant mNAD-MEs. **(A)** Circos representation for co-evolutionary residue positions of α and β mNAD-MEs. Labels in the first and second circles indicate the amino acid number of the α-mNAD-ME from *A. thaliana*. Colored square boxes of the third circle indicate the multiple sequence alignment conservation (from red to cyan, red: the most conserved positions and cyan: the less conserved ones). The fourth circle shows MI scores as histogram. Lines in the center of the circle connect pairs of positions with MI score >6.5. Red lines represent the top 5%; black lines are between 70 and 95%, while gray lines account for the last 70%. Outer red bars indicate regions with the largest number of MI connections, while sequence insertions A, C, D, and F are highlighted in blue. **(B)** Interactive framework showing part of the MI network between residues surrounding, or located in, the sequence insertions. Ovals with letters indicate residues located in insertions A, C, D, and F. **(C)** Interactive framework showing part of the MI network between residues from different subunits. Ovals include residues exhibiting MIs that are shown in the Logo panel. Asterisks indicate the amino acid residue taken as a reference for the exemplified MI network. Logo panels show the amino acid frequency of occurrence along with the corresponding MI value.

FIGURE S3 Exon structure in plant and animal *NAD(P)-MEs*. **(A)** Alignment of the predicted amino acid sequences of all ME family members in *H. sapiens, X. laevis, D. rerio*, and *C. livia*. Fragments encoded by different exons in the amino acid sequence are shown in colors and numbered from E1 to E14. Highlighted sequences indicate mitochondrial targeting peptide. **(B)** Alignment of the predicted amino acid sequences of all ME family members in *P. patens* (three cNADP-ME and one mNAD-ME), *S. moellendorffi* (two cNADP-ME and one mNAD-ME), *A. thaliana* (three cNADP-ME, one plNADP-ME, and two mNAD-ME), and *Z. mays* (three cNADP-ME, two plNADP-ME, and two mNAD-ME). Fragments encoded by different exons in the amino acid sequence are shown in colors and numbered from E0 to E19 for NADP-ME or from e1 to e19 for NAD-ME. Highlighted sequences denote plastidic or mitochondrial targeting peptides.

FIGURE S4 Bayesian 50% majority-rule consensus tree of Class I-ME protein sequences. Evolutionary history was inferred based on the WAG + G + I model. The main lineages are compressed and designated by their name. Nodes with BBP support >0.95 are indicated next to each branch. The area of the triangles representing collapsed clades is not proportional to the number of taxa within the clade. The red section in animal clade represents chromist sequence insertion and the gray section in chromist clade represents fungi sequences.

FIGURE S5 Genes potentially originated from photosynthetic organisms in *R. oryzae* and *M. circinelloides.* Phylogenetic tree using ML method (WAG model) of amino acid sequences. In all cases, numerals indicate boostrap support of branches using 100 replicates. Branch length is proportional to sequence changes. Color line indicates cyanoacteria, red alga, or fungal sequences. These genes were selected based on the photosynthesis-related homologous present in *Phytophthora sojae* (Tyler et al., 2006).

TABLE S1 Accession number of MEF sequences used in this work. ^∗^Indicate sequences used for identity calculation in **Figure 1**. The sequences of MEs, MLEs and OADs were recovered from the National Center for Biotechnology Information database (http://ncbi.nlm.nih.gov). For plants and algal species with entire genome information MEs sequences were extracted from www.phytozome.net, while animal and fungal MEs were retrieved from www.genomesize.com and http://fungidb.org, respectively.
