# Supplementary figures and images for: Chimeric Structure of Plant Malic Enzyme Family: Different Evolutionary Scenarios for NAD- and NADP-Dependent Isoforms
Source: Front Plant Sci. 2018 May 11;9:565. doi: 10.3389/fpls.2018.00565 (PMC5958461; doi:10.3389/fpls.2018.00565)

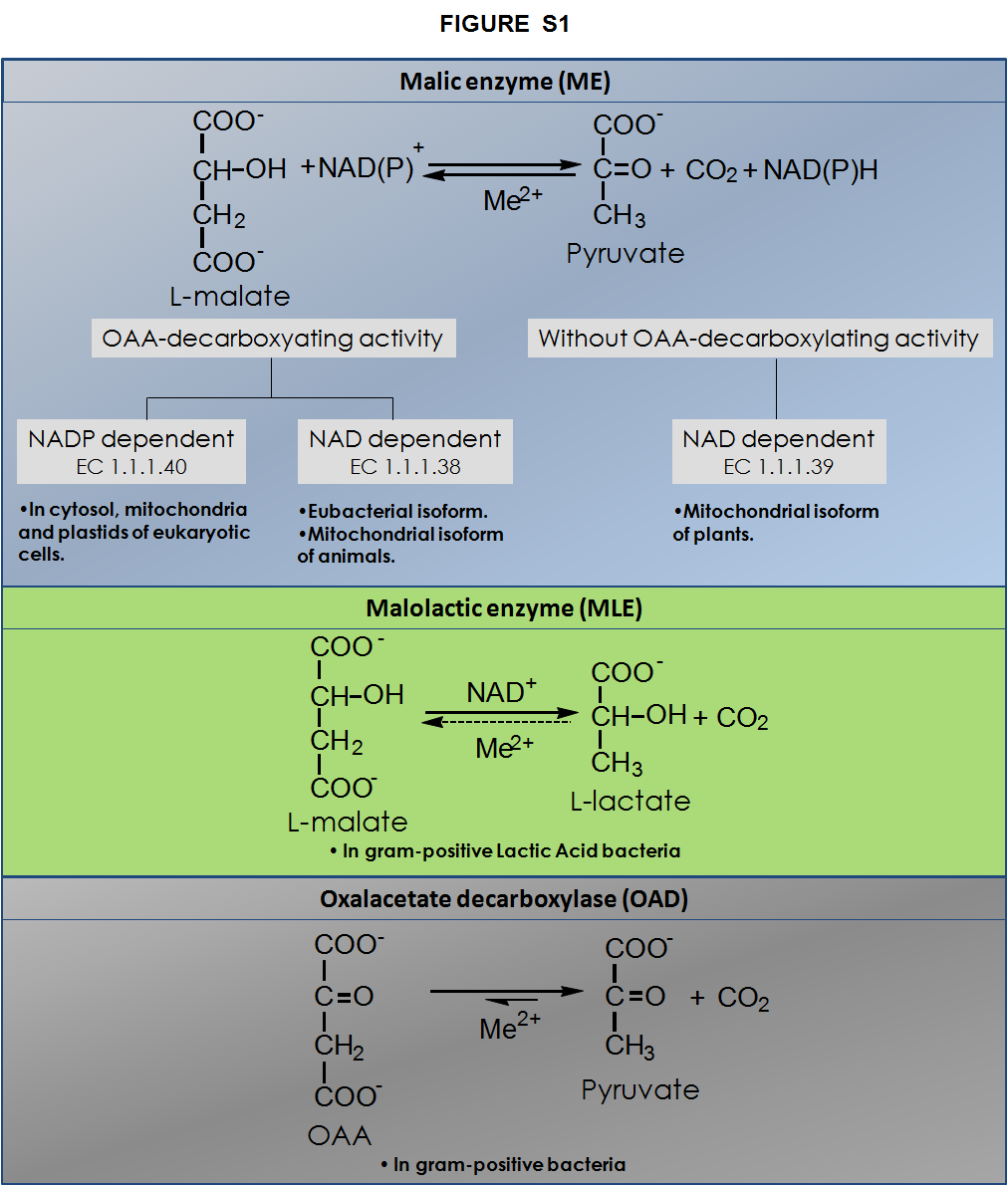

Supplement: FIGURE S1 — Reactions catalyzed by MEF proteins. ME and MLE are involved in the conversion of L-malate into pyruvate and L-lactate, respectively. OAD enzymes catalyze the decarboxylation of OAA to give pyruvate. In the MLE catalysis, NAD acts as cofactor removing two electrons from L-malate and transferring them to pyruvate. Dotted arrow in MLE reaction indicates that the reverse activity has not been measured to date. The presence of a divalent cation (Me2+) is required in all cases. [file Image_1.tif]

FIGURE S2A

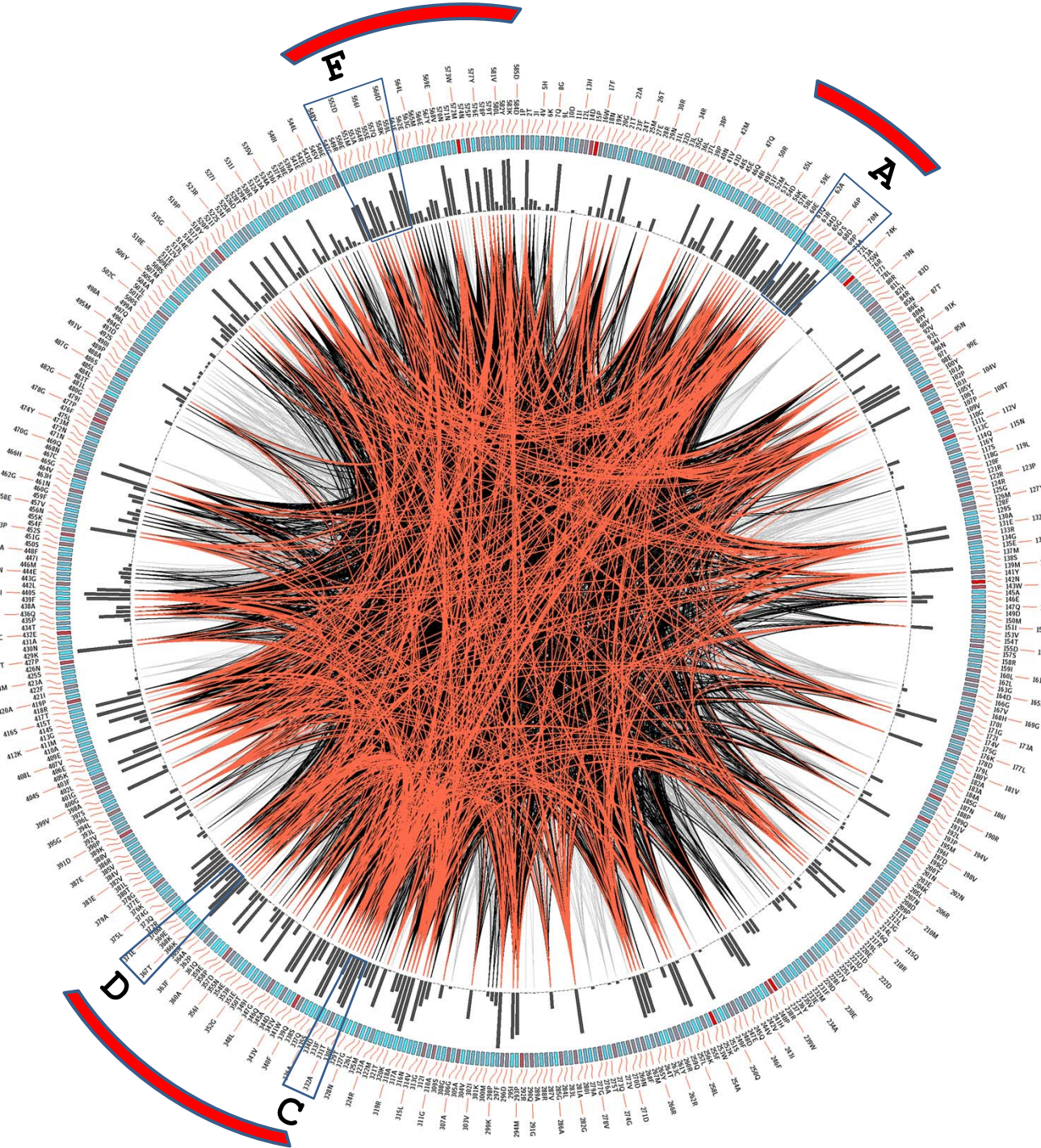

FIGURE S2B

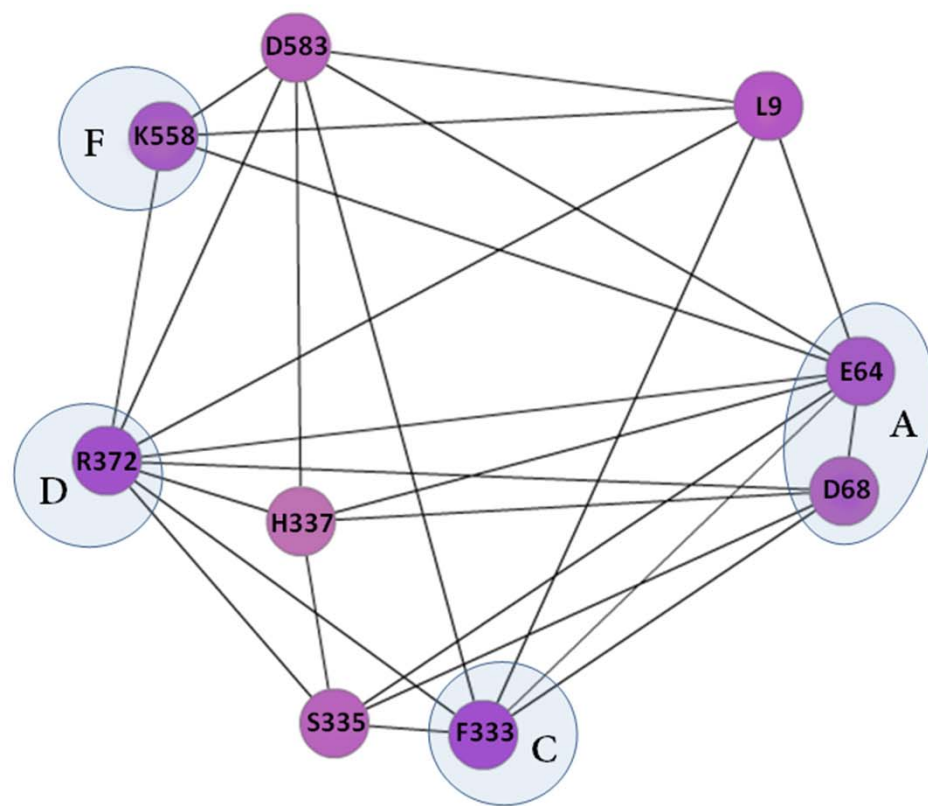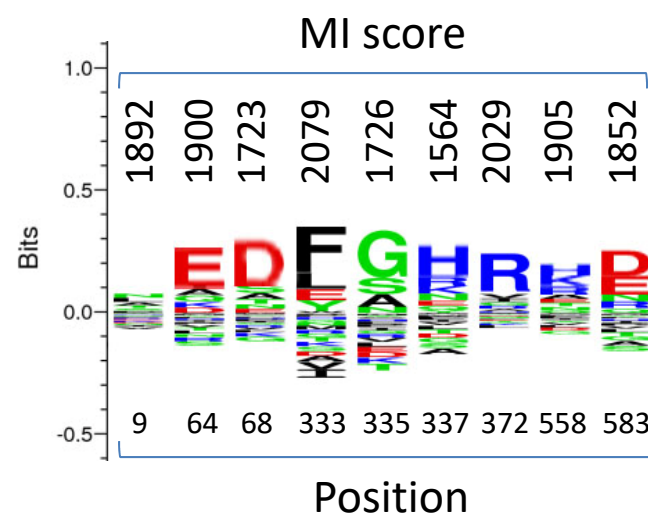

FIGURE S2C

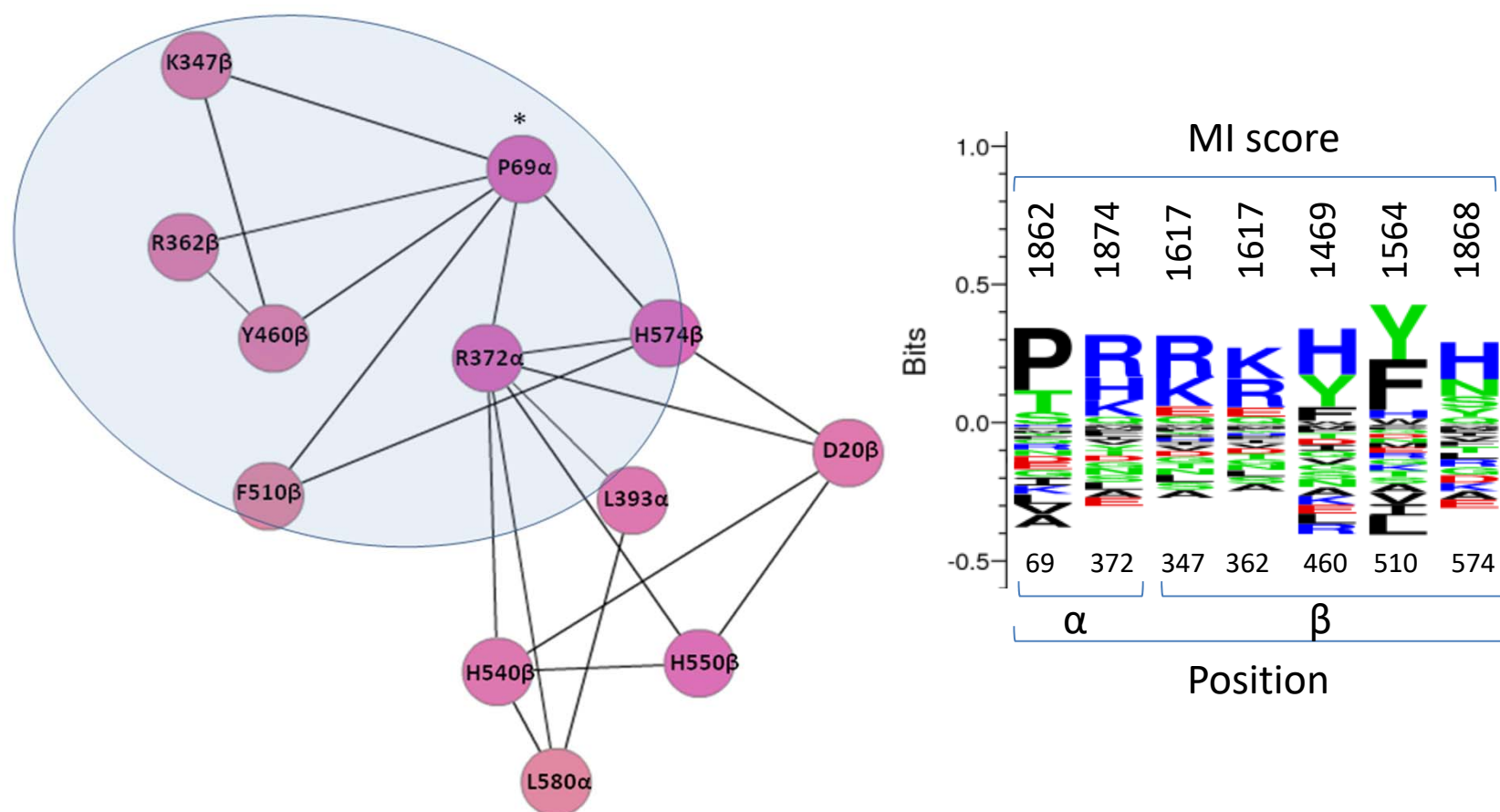

Supplement: FIGURE S2 — Co-evolutionary residue positions of plant mNAD-MEs. (A) Circos representation for co-evolutionary residue positions of α and β mNAD-MEs. Labels in the first and second circles indicate the amino acid number of the α-mNAD-ME from A. thaliana. Colored square boxes of the third circle indicate the multiple sequence alignment conservation (from red to cyan, red: the most conserved positions and cyan: the less conserved ones). The fourth circle shows MI scores as histogram. Lines in the center of the circle connect pairs of positions with MI score >6.5. Red lines represent the top 5%; black lines are between 70 and 95%, while gray lines account for the last 70%. Outer red bars indicate regions with the largest number of MI connections, while sequence insertions A, C, D, and F are highlighted in blue. (B) Interactive framework showing part of the MI network between residues surrounding, or located in, the sequence insertions. Ovals with letters indicate residues located in insertions A, C, D, and F. (C) Interactive framework showing part of the MI network between residues from different subunits. Ovals include residues exhibiting MIs that are shown in the Logo panel. Asterisks indicate the amino acid residue taken as a reference for the exemplified MI network. Logo panels show the amino acid frequency of occurrence along with the corresponding MI value. [file Image_2.pdf]

FIGURE S3A

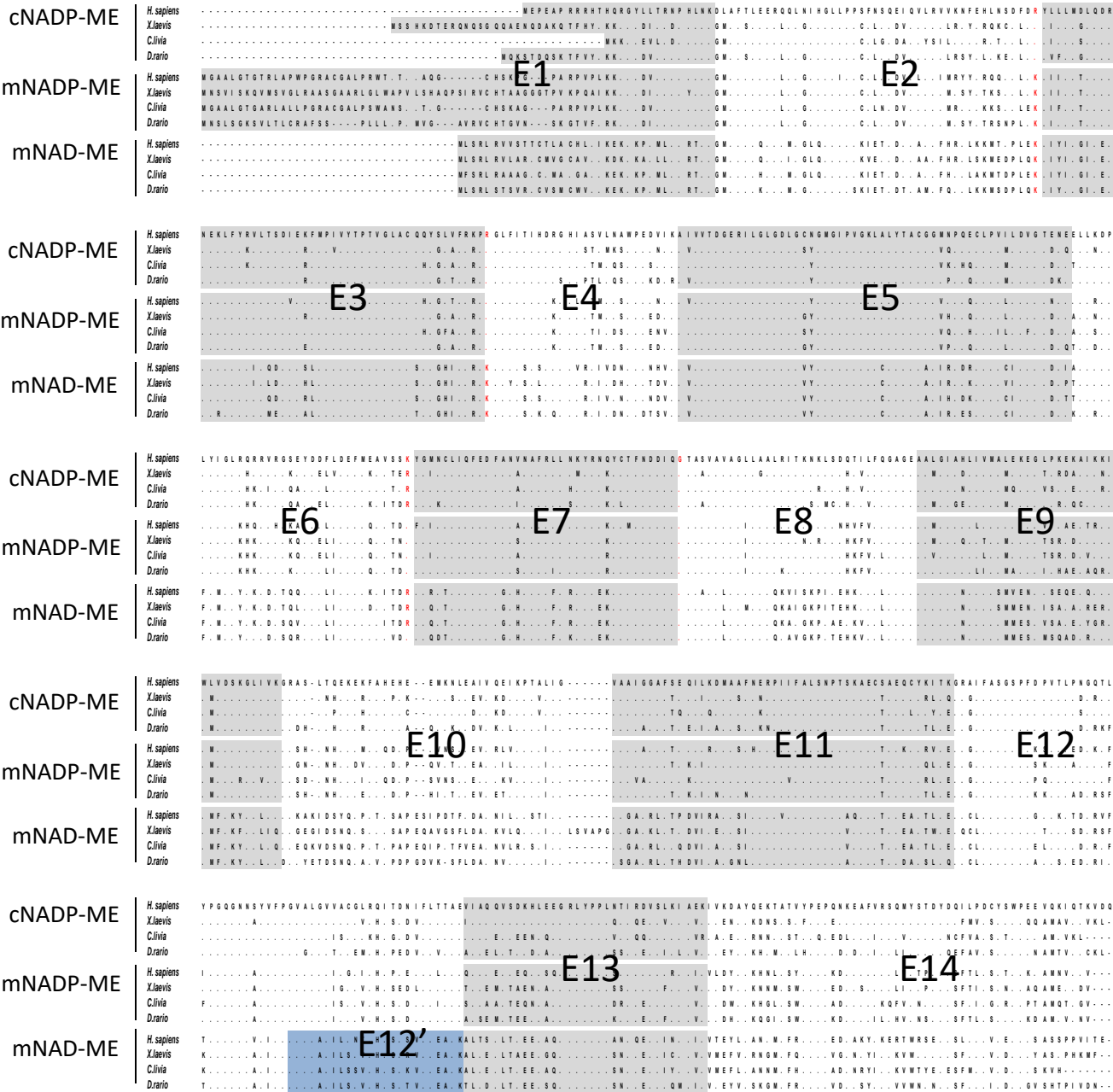

# FIGURE S3B

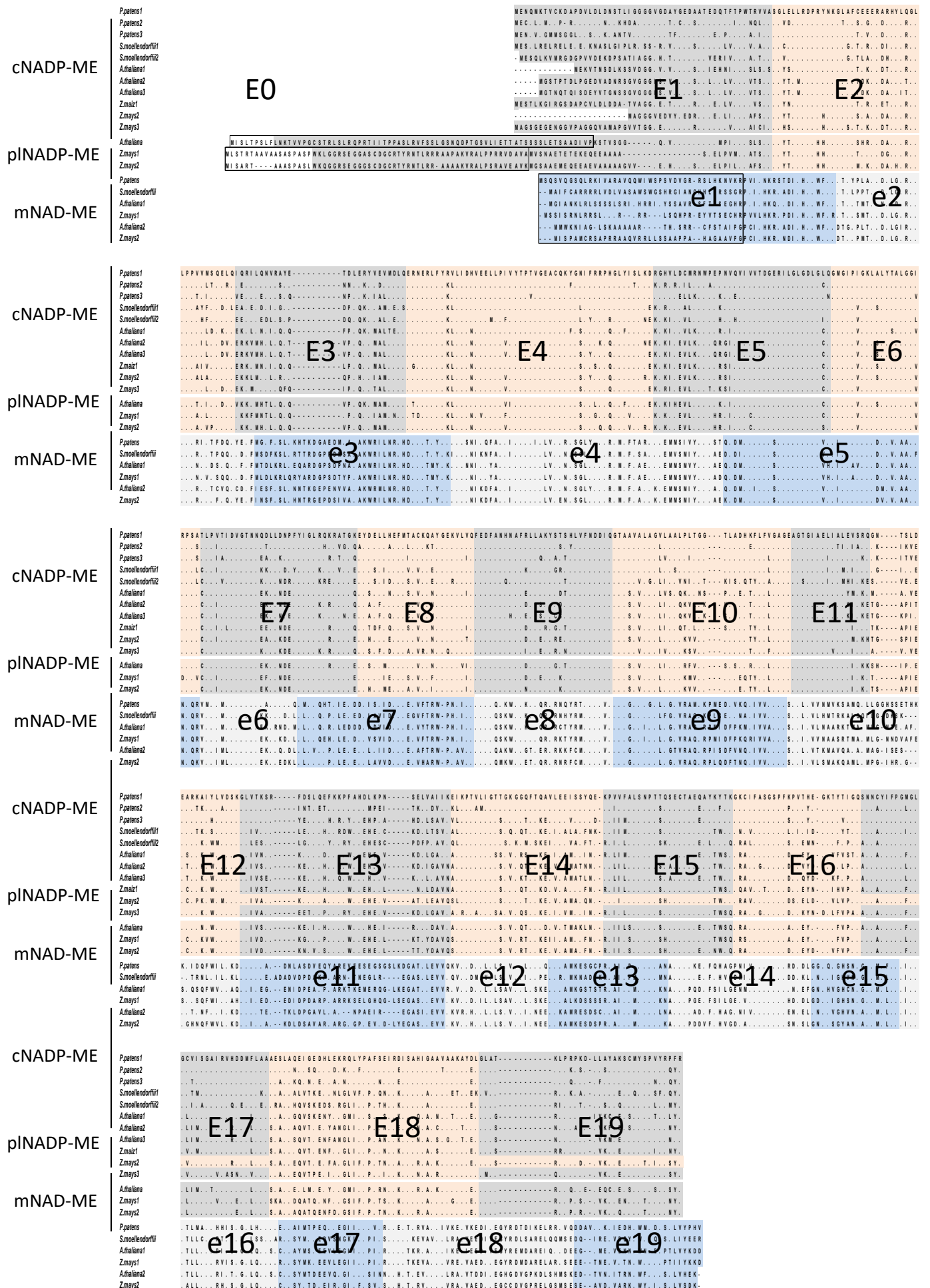

Supplement: FIGURE S3 — Exon structure in plant and animal NAD(P)-MEs. (A) Alignment of the predicted amino acid sequences of all ME family members in H. sapiens, X. laevis, D. rerio, and C. livia. Fragments encoded by different exons in the amino acid sequence are shown in colors and numbered from E1 to E14. Highlighted sequences indicate mitochondrial targeting peptide. (B) Alignment of the predicted amino acid sequences of all ME family members in P. patens (three cNADP-ME and one mNAD-ME), S. moellendorffi (two cNADP-ME and one mNAD-ME), A. thaliana (three cNADP-ME, one plNADP-ME, and two mNAD-ME), and Z. mays (three cNADP-ME, two plNADP-ME, and two mNAD-ME). Fragments encoded by different exons in the amino acid sequence are shown in colors and numbered from E0 to E19 for NADP-ME or from e1 to e19 for NAD-ME. Highlighted sequences denote plastidic or mitochondrial targeting peptides. [file Image_3.pdf]

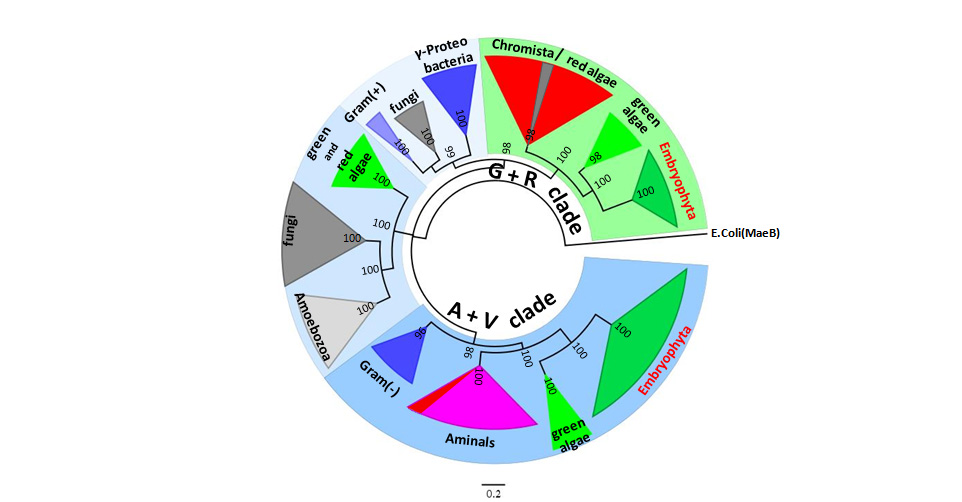

Supplement: FIGURE S4 — Bayesian 50% majority-rule consensus tree of Class I-ME protein sequences. Evolutionary history was inferred based on the WAG + G + I model. The main lineages are compressed and designated by their name. Nodes with BBP support >0.95 are indicated next to each branch. The area of the triangles representing collapsed clades is not proportional to the number of taxa within the clade. The red section in animal clade represents chromist sequence insertion and the gray section in chromist clade represents fungi sequences. [file Image_4.JPEG]

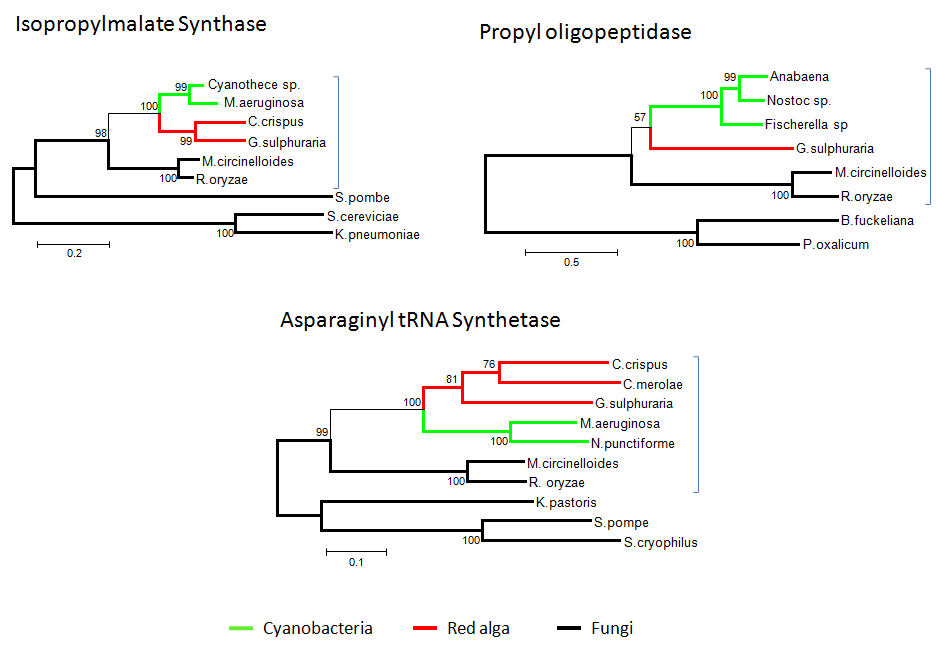

Supplement: FIGURE S5 — Genes potentially originated from photosynthetic organisms in R. oryzae and M. circinelloides. Phylogenetic tree using ML method (WAG model) of amino acid sequences. In all cases, numerals indicate boostrap support of branches using 100 replicates. Branch length is proportional to sequence changes. Color line indicates cyanoacteria, red alga, or fungal sequences. These genes were selected based on the photosynthesis-related homologous present in Phytophthora sojae (Tyler et al., 2006). [file Image_5.TIF]
