# Supplementary material for: Chimeric Structure of Plant Malic Enzyme Family: Different Evolutionary Scenarios for NAD- and NADP-Dependent Isoforms
Source: Front Plant Sci. 2018 May 11;9:565. doi: 10.3389/fpls.2018.00565 (PMC5958461; doi:10.3389/fpls.2018.00565)
Supplement: TABLE S1 — Accession number of MEF sequences used in this work. ∗Indicate sequences used for identity calculation in Figure 1. The sequences of MEs, MLEs and OADs were recovered from the National Center for Biotechnology Information database (http://ncbi.nlm.nih.gov). For plants and algal species with entire genome information MEs sequences were extracted from www.phytozome.net, while animal and fungal MEs were retrieved from www.genomesize.com and http://fungidb.org, respectively. [file Table_1.docx]

| **Supplemental Table 1. Accession number of MEF sequences used in this work** | |
| --- | --- |
| **Eubacteria** | |
| **Gram-negative bacteria** | |
| E.coli (MaeB) | AP_003048.1 |
| E.coli (SfcA) | AP_002102.1* |
| C.freundii | WP_003833011 |
| S.Typhimurium | WP_011233047* |
| K.oxytoca | WP_014229295 |
| C.sakazakii | A7MN74 |
| V.cholerae | A5F1Z0* |
| P.Aeuraginosa | WP_009876884* |
| L.pneumophila | WP_010948656 |
| Mesorhizobium sp. | WP_035705756* |
| T. nitratireducens | AGA34905 |
| D.alkenivorans | ACL02315 |
| D.propionicus | WP_043771052* |
| M.marinus | ABK43288 |
| G.aurantiaca | WP_027524465 |
| D.colitermitum | WP_043589289 |
| O.terrae | WP_012376101* |
| **Gram-positive bacteria** | |
| B. subtilis(YwkA) | P45868* |
| B.megaterium(MEF4) | YP_003597815 |
| B.cereus(MEF3) | ZP_00236573* |
| L.plantarum | NP_784797 |
| E.faecius | ZP_05662292* |
| L.salivarius | YP_536203 |
| S.aureus | YP_416216* |
| L.lactis(MLE) | NP_267056 |
| O.onei(MLE) | YP_810042 |
| L.casei(MLE) | YP_001986787 |
| L.mali(MLE) | WP_003689215 |
| **Archaeplastidia** | |
| **Glaucophyta** | |
| C.paradoxa1 | ConsensusfromContig8102 |
| C.paradoxa2 | ConsensusfromContig38618 |
| **Rhodophyta** | |
| C.crispus1 | XP_005719152 |
| C.rispus2 | XP_005714782 |
| P.purpureum1 | DBLAB_evm.model.contig743.1 |
| P.purpureum2 | DBLAB_evm.model.contig2035.47 |
| C.merolae | XP_005534800 |
| G.sulphuraria | XP_005706775 |
| **Viridiplantae** | |
| **Green algae** | |
| O.lucimarinus1 | fgenesh1_pm.C_Chr_4000073 |
| O.lucimarinus2 | e_gwEuk.6.124.1 |
| O.lucimarinus3 | eugene.0100010014 |
| O.lucimarinus4 | eugene.1200010264 |
| O.lucimarinus5 | eugene.0700010105 |
| O.tauri1 | XP_003080011 |
| O.tauri2 | XP_003078893 |
| O.tauri3 | XP_003082416 |
| O.tauri4 | XP_003080199 |
| O.tauri5 | XP_003074063 |
| M.pusilla1 | MicpuC2.gw1.8.76.1 |
| M.pusilla2 | MicpuC2.fgenesh1_pm.C_scaffold_1000029 |
| M.pusilla3 | MicpuC2.e_gw1.12.58.1 |
| M.pusilla4 | MicpuC2.estExt_fgenesh1_pm.C_110041 |
| C.reinhardtii1 | Cre06.g268750 |
| C.reinhardtii2 | Cre14.g629750 |
| C.reinhardtii3 | Cre14.g629700 |
| C.reinhardtii4 | Cre14.g628650 |
| C.reinhardtii5 | Cre01.g022500 |
| C.reinhardtii6 | Cre06.g251400 |
| C.variabilis1 | XP_005845764 |
| C.variabilis2 | XP_005847663 |
| C.variabilis3 | XP_005850623 |
| **Embryophyta** | |
| **Bryophyta** | |
| P.patensM | Pp3c11_26700* |
| P.patensC1 | Pp3c7_17220* |
| P.patensC2 | Pp3c1_9600 |
| P.patensC3 | Pp3c3_3290 |
| **Lycopodiophyta** | |
| S.moellendorffiiM | Gene176472* |
| S.moellendorffiiC1 | Gene 142026* |
| S.moellendorffiiC2 | Gene130484 |
| **Magnoliophyta (Angiosperms)** | |
| **Eudicot** | |
| A.thalianaC1 | At2g19900 |
| A.thalianaC2 | At5g11670* |
| A.thalianaP | At1g79750* |
| A.thalianaM1 | At2g13560* |
| A.thalianaM2 | At4g00570* |
| V.viniferaC1 | GSVIVG01015311001* |
| V.viniferaC2 | GSVIVG01035291001 |
| V.viniferaP | GSVIVG01016997001* |
| V.viniferaM1 | GSVIVG01018377001* |
| V.viniferaM2 | GSVIVG01026824001* |
| S.lycopersicumM1 | Solyc08g013860 |
| S.lycopersicumM2 | Solyc01g094200 |
| R.communisM1 | 29709.t000046 |
| R.communisM2 | 30174.t000385 |
| M.truncatulaM1 | Medtr8g009500 |
| M.truncatulaM2 | Medtr8g032040 |
| A.hypochondriacus | U01162 |
| **Monocot** | |
| Z.maizC1 | GRMZM2G085019* |
| Z.maizC2 | GRMZM5G886257 |
| Z.maizC3 | GRMZM2G118770 |
| Z.maizP1 | GRMZM2G085019 |
| Z.maizP2 | GRMZM2G122479* |
| Z.maizM1 | GRMZM2G406672* |
| Z.maizM2 | GRMZM2G085747* |
| O.sativaC1 | Os01g52500* |
| O.sativaC2 | Os05g09440 |
| O.sativaC3 | Os01g54030 |
| O.sativaP | Os01g09320* |
| O.sativaM1 | Os07g31380* |
| O.sativaM2 | Os10g35960* |
| S.bicolorM1 | Sobic.002G309400 |
| S.bicolorM2 | Sobic.001G201700 |
| S.italicaM1 | Si029215m |
| S.italicaM2 | Si034747m |
| A.tauchiiM1 | EMT06598 |
| A.tauchiiM2 | EMT10922 |
| P.virgatumM1 | Pavirv00022665 |
| P.virgatumM2 | Pavirv00010249 |
| **Chromista** | |
| **Heterokonta** | |
| **Oomycetes** | |
| P.sojae1 | XP_009525804 |
| P.sojae2 | XP_009525804 |
| P.infestans1 | XP_002896607 |
| P.infestans2 | XP_002901849 |
| A.laibachii1 | CCA15917 |
| A.laibachii2 | CCA25004 |
| **Diatoms** | |
| T.oceanica | EJK44344 |
| T.pseudonana | BAO52744 |
| P.tricornutum | XP_002177890 |
| **Pelagophyceae** | |
| A.anophagefferens | XP_009033200 |
| **Phaeophyceae** | |
| E.siliculosus1 | D7FW76 |
| E.siliculosus2 | CBN77654 |
| **Hatophyta** | |
| E.hukleyi | XP_005760562 |
| **Cryphothyta** | |
| G.theta1 | XP_005842105 |
| G.theta2 | XP_005831287 |
| **Chlorarachniophyta (Rhizaria)** | |
| B.natans1 | aug1.4_g1863 |
| B.natans2 | fgenesh1_pg.56_#_131 |
| **Opisthokont** | |
| **Fungi** | |
| A.oryzae1 | XP_001826448 |
| A.oryzae2 | XP_001825515 |
| B.cinerea | EDN30340 |
| P. chrysogenum1 | CAP91520 |
| P. chrysogenum2 | CAP96922 |
| R.oryzae1 | RO3G_08533 |
| R.oryzae2 | RO3G_04512 |
| R.oryzae3 | RO3G_09392 |
| R.oryzae4 | RO3G_05452 |
| M.circinelloides1 | jgilMucci2l78524lMucci1.fgeneshMC_pg.2_*_816 |
| M.circinelloides2 | jgilMucci2l11639lMucci1.gw1.4.250.1 |
| M.circinelloides3 | jgilMucci2l166127lfgenesh1_pg.07_*_620 |
| M.circinelloides4 | gilMucci2l186772lestExt_fgenesh1_pm.C_030371 |
| N.frontalis | P78715 |
| P.pastoris | CAY70158 |
| C.tropicalis | EER34057 |
| S.pompe | P40375 |
| S.japonicum | EEB07095 |
| S.cerevisiae | EEU06589 |
| C.gatti | XP_003194651 |
| P.carnosa | XP_007391046 |
| **Animalia** | |
| A.suum | P27443 |
| B.malayi | XP_001902120 |
| C.elegans | NP_496968 |
| T.spiralis | XP_003379252 |
| D.rerioC | NP_001296966* |
| D.rerioM1 | NP_001082825* |
| D.rerioM2 | NP_001003627* |
| X.laevisC | XP_002938500* |
| X.laevisM1 | NP_001088519* |
| X.laevisM2 | NP_001082582* |
| C.liviaC | NP_001269745* |
| C.liviaM1 | XP_005510791* |
| C.liviaM2 | XP_005503027* |
| H.sapiensC | NP_002386* |
| H.sapiensM1 | NP_006671* |
| H.sapiensM2 | NP_002387* |
| **Amoebozoa** | |
| D.purpureum | jgi\|Dicpu1\|44565\|estExt_Genewise1.C_20112 |
| D.discoideum | Q6TU48 |
| P.palladium | EFA83632 |
| D.fasciculatum | XP_004355233 |
| M.balamuthi | AAN86690 |
